# Supplementary material for: Comparative genomics provides new insights into the diversity, physiology, and sexuality of the only industrially exploited tremellomycete: Phaffia rhodozyma
Source: BMC Genomics. 2016 Nov 9;17:901. doi: 10.1186/s12864-016-3244-7 (PMC5103461; doi:10.1186/s12864-016-3244-7)
Supplement: Additional file 6: — List of orphan genes with links to PFAM (related to Additional file 1: Table S1). (ZIP 1428 kb) [file 12864_2016_3244_MOESM6_ESM.zip › PFAM_UNIQUE/G05355_P.html]

#                                                                            --- full sequence --- -------------- this domain -------------   hmm coord   ali coord   env coord
  
# target name        accession   tlen query name           accession   qlen   E-value  score  bias   #  of  c-Evalue  i-Evalue  score  bias  from    to  from    to  from    to  acc description of target
  
#------------------- ---------- ----- -------------------- ---------- ----- --------- ------ ----- --- --- --------- --------- ------ ----- ----- ----- ----- ----- ----- ----- ---- ---------------------
  
G05355\_P             -             68 Activator\_LAG-3      PF11498.3    468       6.3    4.1  14.9   1   1     0.015       6.9    4.0  14.9   406   427    14    32     4    44 0.53 - Transcriptional activator LAG-3  
G05355\_P             -             68 APC\_CDC26            PF10471.4     79     0.062   12.7   5.4   1   1   9.3e-05     0.069   12.6   5.4    24    60    12    44     7    65 0.52 - Anaphase-promoting complex APC subunit 1  
G05355\_P             -             68 Caudo\_TAP            PF02413.12   130       0.1   11.4   1.9   1   1   6.9e-05       0.1   11.4   1.9    48    86     3    42     1    59 0.79 - Caudovirales tail fibre assembly protein  
G05355\_P             -             68 dsDNA\_bind           PF01984.15   107     0.048   12.3   3.1   1   1   4.6e-05     0.055   12.1   3.1    16    46    12    42     3    56 0.69 - Double-stranded DNA-binding domain  
G05355\_P             -             68 DUF1151              PF06625.6    122     0.081   11.3   1.8   1   1   5.5e-05     0.082   11.3   1.8    48    87     2    39     1    58 0.81 - Protein of unknown function (DUF1151)  
G05355\_P             -             68 DUF1525              PF07511.6    114      0.28    9.9   2.8   1   1   0.00012      0.36    9.5   2.8    34    71     6    44     3    51 0.82 - Protein of unknown function (DUF1525)  
G05355\_P             -             68 DUF2489              PF10675.4    131       4.1    5.8   6.2   1   1    0.0018       5.3    5.4   6.2    13    32     9    28     5    39 0.53 - Protein of unknown function (DUF2489)  
G05355\_P             -             68 DUF2547              PF10818.3     94      0.32   10.3   3.0   1   1   0.00024      0.36   10.2   3.0    48    75    14    40     3    50 0.51 - Protein of unknown function (DUF2547)  
G05355\_P             -             68 DUF2681              PF10883.3     87      0.25   10.3   3.4   1   1   0.00015      0.31   10.0   3.4    33    64     9    40     5    46 0.84 - Protein of unknown function (DUF2681)  
G05355\_P             -             68 DUF2722              PF10846.3    417     0.085   10.5   2.5   1   1   0.00021     0.086   10.5   2.5    18    47    12    47     1    54 0.49 - Protein of unknown function (DUF2722)  
G05355\_P             -             68 DUF2756              PF10956.3    104       2.7    7.0   6.5   1   1    0.0015         3    6.8   6.5    56    71    14    29     2    54 0.51 - Protein of unknown function (DUF2756)  
G05355\_P             -             68 DUF2802              PF10975.3     70       2.8    6.5   6.0   1   1    0.0025       3.7    6.1   6.0    14    33    11    28     5    40 0.49 - Protein of unknown function (DUF2802)  
G05355\_P             -             68 DUF3523              PF12037.3    276         1    6.9   6.0   1   1    0.0018       1.2    6.7   6.0   123   150    10    37     4    46 0.75 - Domain of unknown function (DUF3523)  
G05355\_P             -             68 DUF3584              PF12128.3   1201      0.18    7.8   1.9   1   1    0.0013      0.18    7.7   1.9   740   776    10    46     2    56 0.68 - Protein of unknown function (DUF3584)  
G05355\_P             -             68 DUF3947              PF13135.1     76       3.5    6.6   7.5   1   1    0.0014       4.1    6.4   7.5    33    52    15    31     9    46 0.54 - Protein of unknown function (DUF3947)  
G05355\_P             -             68 DUF4175              PF13779.1    820      0.24    7.7   5.2   1   1   0.00067      0.24    7.7   5.2   518   542    13    36     2    51 0.52 - Domain of unknown function (DUF4175)  
G05355\_P             -             68 DUF4207              PF13904.1    264       7.2    4.6   7.5   1   1     0.016       8.6    4.3   7.5   195   217    14    36     8    43 0.59 - Domain of unknown function (DUF4207)  
G05355\_P             -             68 Dynactin             PF12455.3    274      0.34    8.4   4.0   1   1   0.00028      0.42    8.1   4.0     2    40    11    45    10    49 0.82 - Dynein associated protein  
G05355\_P             -             68 E\_Pc\_C               PF06752.7    230       1.5    6.9   5.0   1   1    0.0011       1.3    7.1   5.0    24    45    15    36     7    65 0.62 - Enhancer of Polycomb C-terminus  
G05355\_P             -             68 FimP                 PF09766.4    355       3.1    5.4   6.7   1   1    0.0057       3.4    5.3   6.7   221   242    12    33     2    46 0.44 - Fms-interacting protein  
G05355\_P             -             68 Med15                PF09606.5    799      0.14    9.0   6.1   1   1   0.00065      0.12    9.1   6.1   366   384    14    32     2    59 0.51 - ARC105 or Med15 subunit of Mediator complex non-fungal  
G05355\_P             -             68 Med25\_NR-box         PF11244.3     89       6.7    5.9  12.1   1   1    0.0029       8.6    5.6  12.1    10    29    10    30     3    37 0.55 - Mediator complex subunit 25 C-terminal NR box-containing  
G05355\_P             -             68 Med3                 PF11593.3    379       3.3    5.5   9.3   1   1     0.011       3.7    5.3   9.3   333   350    12    29     3    42 0.45 - Mediator complex subunit 3 fungal  
G05355\_P             -             68 Med9                 PF07544.8     83      0.78    8.2   5.4   1   1    0.0013      0.98    7.9   5.4    46    82     3    38     1    39 0.80 - RNA polymerase II transcription mediator complex subunit 9  
G05355\_P             -             68 OmpH                 PF03938.9    158      0.32    9.6   2.9   1   1    0.0019      0.34    9.5   2.9    94   118    13    38     4    53 0.52 - Outer membrane protein (OmpH-like)  
G05355\_P             -             68 PAT1                 PF09770.4    808      0.23    8.3   2.9   1   1    0.0014      0.21    8.4   2.9   322   340    14    32     2    61 0.47 - Topoisomerase II-associated protein PAT1  
G05355\_P             -             68 PKK                  PF12474.3    142      0.34    9.3   3.3   1   1   0.00033      0.39    9.1   3.3    91   120    10    39     3    48 0.68 - Polo kinase kinase  
G05355\_P             -             68 SprA-related         PF12118.3    295       4.7    5.1   6.5   1   1     0.037       5.1    5.0   6.5   125   138    14    27     2    45 0.44 - SprA-related family  
G05355\_P             -             68 Trichoplein          PF13868.1    350       2.5    5.0   7.5   1   1    0.0058       2.9    4.9   7.5   271   297    12    38     7    44 0.64 - Tumour suppressor, Mitostatin  
G05355\_P             -             68 TSA                  PF03249.8    503       1.8    5.6   5.8   1   1   0.00032       1.9    5.6   5.8   306   329    11    34     2    50 0.63 - Type specific antigen  
G05355\_P             -             68 Vicilin\_N            PF04702.7    147       5.9    5.2   6.9   1   1    0.0023       6.8    5.0   6.9    99   114    14    29     4    44 0.44 - Vicilin N terminal region
